# Telehealth: A Useful Tool for the Management of Nutrition and Exercise Programs in Pediatric Obesity in the COVID-19 Era

**DOI:** 10.3390/nu13113689

**Published:** 2021-10-20

**Authors:** Valeria Calcaterra, Elvira Verduci, Matteo Vandoni, Virginia Rossi, Elisabetta Di Profio, Vittoria Carnevale Pellino, Valeria Tranfaglia, Martina Chiara Pascuzzi, Barbara Borsani, Alessandra Bosetti, Gianvincenzo Zuccotti

**Affiliations:** 1 Pediatric and Adolescent Unit, Department of Internal Medicine, University of Pavia, 27100 Pavia, Italy; 2 Pediatric Department, “Vittore Buzzi” Children’s Hospital, 20154 Milan, Italy; elvira.verduci@unimi.it (E.V.); virginia.rossi@unimi.it (V.R.); elisabetta.diprofio@unimi.it (E.D.P.); valeria.tranfaglia@unimi.it (V.T.); martina.pascuzzi@unimi.it (M.C.P.); barbara.borsani@unimi.it (B.B.); alessandra.bosetti@asst-fbf-sacco.it (A.B.); gianvincenzo.zuccotti@unimi.it (G.Z.); 3 Department of Health Sciences, University of Milan, 20142 Milan, Italy; 4 Laboratory of Adapted Motor Activity (LAMA), Department of Public Health, Experimental Medicine and Forensic Science, University of Pavia, 27100 Pavia, Italy; matteo.vandoni@unipv.it (M.V.); vittoria.carnevalepellino@unipv.it (V.C.P.); 5 Pediatric Clinical Research Center Romeo ed Enrica Invernizzi, Department of Biomedical and Clinical Science “L. Sacco”, University of Milan, 20157 Milan, Italy

**Keywords:** telehealth, obesity, tele-exercise, tele-nutrition, COVID-19, children, pediatrics

## Abstract

The COVID-19 pandemic has led to the implementation of policies that mandate various restrictions on daily life, including social distancing, the closure of public services and schools, and movement limitations. Even though these restrictive measures decreased the COVID-19 spread, they may have detrimental effects on various lifestyle components such as physical inactivity, sedentary behavior, and dietary habits, influencing the maintenance of weight and contributing to obesity among children and adolescents. The coexistence of childhood obesity and COVID-19 and changes in the bioecological environment have put children and adolescents at increased risk for developing obesity and exacerbating the severity of this disorder. The use of telehealth technology is a modern approach useful for the delivery of health care services by health care professionals, where distance is a critical factor. Telehealth is effective in promoting increased self-monitoring and behavioral change, and provides the opportunity to perform online nutritional support and exercise training programs to promote a healthy lifestyle and reduce sedentary behaviors in children and adolescents. Telehealth, including tele-exercise and tele-nutrition, has the potential to address many of the key challenges in providing health services, including in patients with obesity during the COVID-19 outbreak. This narrative review aims to describe the role of telehealth as an opportunity in the management of pediatric obesity in the COVID-19 era, and to deliver nutrition and exercise programs for the maintenance of health.

## 1. Introduction

A global pandemic caused by the new coronavirus (COVID-19) has led to the implementation of policies that mandate various restrictions on daily life, including social distancing, the closure of public services and schools, and movement limitations [1]. Even though these restrictive measures decreased the COVID-19 spread, they may have detrimental effects on various lifestyle components and lead to physical inactivity, sedentary behaviors, and dietary habits, thereby influencing the maintenance of weight and contributing to obesity among children and adolescents [1,2,3]. Significant changes in dietary consumption patterns, frequency of meals, and skipping breakfast have been described [2,3]. Additionally, a decreased frequency of engaging in active transport, moderate or vigorous and high-intensity housework, physical activity (PA) during leisure time, and walking during leisure time, as well as increased sedentary, sleeping, and screen time, have been reported [4]. 

The consequences of these changes are even more serious because an active lifestyle contributes to the physical and mental health of children and adolescents, including a more effective immune system [5]. Sixty minutes/day of moderate to vigorous intensity PA participation for girls and boys aged 5–17 is recommended to achieve health and psychological benefits [6]. During the COVID-19 pandemic, PA or exercise were restricted, leading people to sedentary behavior and weight gain.

Telehealth is a set of services available to patients in specific geographic locations where the health care professional uses interactive audio and video telecommunication systems that permit asynchronous or real-time two-way communication between the patient and distant-site clinician [7]. The use of telehealth technology is a modern approach useful for the delivery of health care services by health care professionals where distance is a critical factor, using information and communication technologies (ICT) for the exchange of information [7]. As reported, telehealth and mobile health (mHealth) technologies are effective tools for obesity management [7].

Telehealth is effective in promoting increased self-monitoring, behavioral change, and weight reduction and it provides the opportunity to perform online nutritional support and exercise training programs to promote a healthy lifestyle and reduce sedentary behaviors in children and adolescents [8]. The WHO Regional Office for Europe has provided advice for health care providers on PA maintenance during isolation that includes the support of online resources [9].

**Figure 1:**
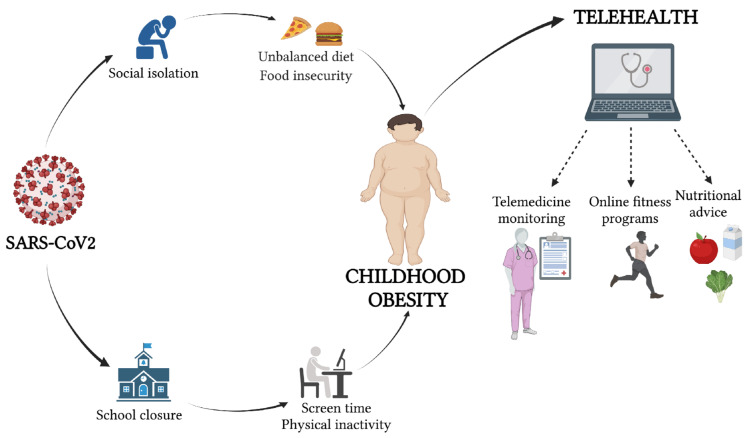
The role of telehealth during the COVID-19 era (created with BioRender.com, accessed on 16 October 2021).

Telehealth has the potential to address many of the key challenges in providing health services during the outbreak of COVID-19 in patients with obesity, too (Figure 1). 

This manuscript provides an in-depth narrative review of how COVID-19 interfered with the physical activity routine and diet in girls and boys with obesity, increasing the risk factors related to pathology and exacerbating the severity of illness. We described the role of telehealth as an effective method to spread knowledge on food and exercise habits as well as an opportunity to manage and formulate interventions in obese children and adolescents during the COVID-19 pandemic.

## 2. Methods

We performed a narrative review [9]. The most relevant published studies (original papers, metanalysis, clinical trials, and reviews) in the English literature in the past 15 years were considered. The following search keywords were used: childhood obesity, pediatric obesity; children; telehealth; telemedicine; diet; sedentary; physical activity; COVID-19. PubMed, Scopus, EMBASE, and Web of Science were used as electronic databases for research. The contributions were collected, and the resulting draft was discussed before finalizing. The final version was approved by all the coauthors.

## 3. Telehealth and the COVID-19 Era

In December 2019, the SARS-CoV-2 novel coronavirus and its related disease, COVID-19, was discovered and identified in Wuhan, China. On 11 March 2020, COVID-19 became a World Health Organization-declared global pandemic owing to its rapid spread around the globe, having infected approximately 212 million people by 23 August 2021 [10,11,12,13,14,15,16].

Several strategies have been implemented to minimize the spread of the virus as much as possible, particularly social distancing and home quarantine policies. However, these strategies, while necessary, have resulted not only in the disruption of people’s normal routines, but also in significant challenges to our societies in almost every area, including health care. In particular, we report limited capacity to meet demand in outpatient settings, such as pediatricians’ private practices and hospital clinics; postponement of elective surgeries, procedures, and imaging studies; recommendations to avoid the emergency room by children and families who may need health care; and missed appointments for routine child immunizations [17].

The current global COVID-19 pandemic is underscoring the urgency of leveraging digital approaches to optimize pediatric health care delivery during this pandemic era. As the restrictions and limitations on in-person or face-to-face visits continue, many patients, families, and physicians, including pediatricians, are increasingly recognizing the potential of telemedicine [17,18,19]. The emergent situation due to COVID-19 created a challenge and, simultaneously, an opportunity to continue providing pediatric clinical care. Continued pediatric health care has resulted from the enhancement of telemedicine disciplines, reduction of geographic barriers, and increased flexibility in scheduling and the type of services offered [20]. Virtual clinical visits are able to manage routine clinical care and increased demand for the same in the event of clinic closures [21]. During the COVID-19 pandemic, virtual visits also allow patients to be connected to testing resources while ensuring that self-quarantine obligations are observed [21,22,23,24].

The terms telehealth and telemedicine are usually employed interchangeably, but telehealth has developed to encapsulate a wider range of digital health care activities and services [25]. In fact, where telemedicine is specifically referred to as the practice of medicine by remote means, telehealth is an inclusive expression that includes all health care and health system components and activities that are conducted through telecommunications technology [26,27,28,29].

Several technologies can be leveraged to access telemedicine services, including mHealth (or mobile health), video and audio technologies, digital photography, store-and-forward, and remote patient monitoring.

mHealth refers to the health apps and programs that patients use on their smartphones, tablets, or laptops through apps that allow patients to track health measurements, set medication, and appointment reminders, and share information with physicians [26]. Store-and-forward telehealth refers to the capture, storage, and transmission of patient health information, such as CT scans, MRIs, X-rays, and other data, for the delivery of health care using data storage and transmission technology that will be used to assess patients and assist them in treatment through secure and reliable technologies. Finally, RPM refers to the use of telemedicine measurement devices, such as smartphone cameras, digital stethoscopes, ophthalmoscopes, otoscopes, and wearable biosensors, to record and transmit data to health care professionals, representing a further evolution of telemedicine [26,27]. Wearables and other electronic monitoring devices are used to collect and transfer vital sign data, including blood pressure, heart statistics, oxygen levels and breathing rates [26].

Indeed, telemedicine services are not new to the global health care community, yet the rate of access to these services remains low. However, in response to the current emergency situation driven by the COVID-19 pandemic, governments and health systems are encouraging the use of telemedicine. The rapid progression of telemedicine and the rising demand for this service as we face the current COVID-19 pandemic make it imperative that telemedicine itself adapts to the needs of patients and their families during the COVID-19 pandemic [27].

The use of telemedicine facilities has had a positive impact in a number of ways during the current public health emergency [22]. In addition to facilitating triage, performed before patients visit an emergency department, whether for COVID-19 respiratory symptoms or other symptoms, telemedicine has also allowed the rapid deployment of large numbers of health care providers and the provision of services when local hospitals and health centers are unable to meet demand. Therefore, telemedicine services have provided much needed health care not only to infected people but also to uninfected people during this infectious pandemic [30]. Indeed, telemedicine has the great advantage of allowing patients with mild illnesses to have the supportive care they need, while minimizing their exposure to other patients with acute illnesses.

**Figure 2:**
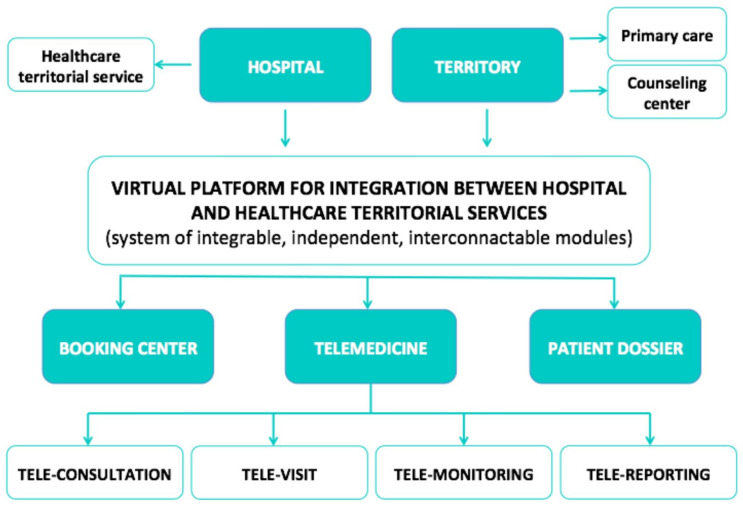
Overview of the COD 20 system: virtual platform for integration between hospital and healthcare services, reported by Zuccotti et al. [25] (created with BioRender.com, accessed on 16 October 2021).

Zuccotti et al. recently reported an Italian project about the implementation of active domiciliary monitoring (COD19) and a homecare hospital system (COD20) in COVID-19 patients, supported by the certified Amazon Web Services Serverless platform [25]. This monitoring system provides continuous assessment of critical medical status, detection of socially and clinically relevant issues, and the supply of necessary clinical services as a telemedicine service. COD20 is an interactive patient-to-physician video consultation system that enables an accurate evaluation of the clinical status and the potential need for any further visit, prioritizing outpatient in presence consultations or defining them as manageable through the new video consultation model [25]. This service was readily needed during COD19 in response to the identification of comorbidities in the monitored patients, but it also provides a significant management facility after the lockdown. The COD20 system supports the integration between the hospital and healthcare territorial system, and automates and simplifies the appointment booking process through online connection with the regional booking center [25] (Figure 2). The visit is provided by video-consultation using web-based technologies, not requiring any configuration of the patient’s personal devices. Following the video-consultation, the report is stored in the file for possible future reporting and publication in the electronic health record [25]. As reported by the authors [25], the model was well used during the pandemic crisis. Over 58 days of activity, the service took charge of 1097 patients and managed a total of 27,195 calls. A total of 38% of the patients were referred to the service following discharge from a hospital unit (38%), an emergency department 40%, or an occupational medicine service (22%). The disease course had a positive outcome, except for 52 patients under surveillance that were admitted to hospital following detection of pathological parameters during consultation. Overall, the service was well accepted by the patients. Only 1.6% of the patients responding to the satisfaction questionaire gave at least one negative rating of the relationship with the care provision, ease of measuring the clinical parameters, or level of general satisfaction with the service [25].

Remotely delivered patient visits are particularly valuable in the field of pediatrics, as patients and their families often have to deal with impediments, such as a limited number of pediatric specialists and barriers to long-distance travel [31,32,33,34]. However, recent improvements in pediatric telemedicine have made it possible to provide pediatric services to medically underserved regions and low-income countries [35,36,37].

As stated before, the onset of the COVID-19 pandemic has significantly affected traditional health care delivery systems. It has been necessary to implement social distancing, but, at the same time, it has been important not to suspend continuity of care [17,22,24,32,38,39,40]. Therefore, it has been of paramount importance to implement all those innovative services that ensure the delivery of remote health care visits and pediatric services [41].

A study undertaken by Walters et al. [42] at Cincinnati Children’s Hospital evidenced that, through the provision of telemedicine, children received care in the most appropriate and cost-effective way for mild acute problems, offering the right care, at the right time, in the right place [42]. The research examined telemedicine visits during five months in the pandemic period: 62% of the visits were for acute problems, such as rash, eye drainage/redness, constipation, and cough, while 38% were for chronic conditions, in particular mental health problems (including ADHD) and asthma [42]. This study found that the implementation of telemedicine into pediatric primary care was feasible even for children from underserved communities and families reported that telemedicine provided care, guaranteeing to avoid unnecessary emergency department/urgency care visits. However, among the clinical population, Walters et al. [42] saw racial disparities in the use of telemedicine; that is, Black families used telemedicine significantly less than non-Black families, as well as non-English speaking families, who did not have access to the telemedicine visits owing to Cincinnati Children Hospital technological constraints. These types of disparities are consistent with previous literature suggesting that technology can widen inequalities [32,42,43,44]. 

Capusan et al. [45] evaluated the application of telemedicine to a group of children with pulmonary disease; following the visits, a questionnaire regarding the experience of telemedicine was administered to parents with questions related to technology (i.e., type of device used, platform used, and so on), visit, overall satisfaction, and likelihood of using the telemedicine platform again. The survey results indicated high overall satisfaction with telemedicine: 82% of participants strongly agreed or agreed that they would use telemedicine services again. Two main limitations were identified: a low response rate to the questionnaire (50/281, about 18%) and the short duration of the survey, which lasted only one month [45].

Another area where the use of telemedicine during the COVID-19 pandemic has been beneficial is pediatric sleep care, as observed in a case report of a 12-year-old male with nonverbal autism and morbid obesity [46]. Through telemedicine consultation and home sleep apnea testing, the patient was diagnosed with obstructive sleep apnea and an irregular sleep–wake disorder; this unique use of the health care system in the care of a complex patient with multiple sleep disorders demonstrates the utility of remote care and testing [46].

Therefore, the implementation of telemedicine is especially useful for patients with chronic disease, such as obesity, which demands ongoing management. Withdrawal of visits for the management and care of chronic obesity delays treatment and may lead to increased disease burden and poor outcomes [47,48,49]. Clinicians have well recognized the importance of maintaining continuity of care in obese patients, who are at higher risk for COVID-19 disease severity, which may result from factors directly attributable to the pathophysiology of obesity and/or chronic obesity comorbidities [41,50].

Specifically, O’Hara et al. [41] conducted a study that evaluated the application of telemedicine for obesity care management during the COVID-19 pandemic. It is of major importance to teach patients and parents how to manage such chronic disease remotely. For example, it has been possible to keep track of data, accurately taken, such as vital parameters and patients’ weight, which play a diriment role in the management of the obese patient. In addition, tools such as the glucometer and blood pressure cuff have proven to be useful and reliable [41]. 

In addition, Fleishman et al. [51] and Rhodes et al. [52] have also examined the positive impact of telemedicine on weight management, confirmed through a significant post-treatment decrease in BMI and reduced post-treatment total caloric intake through a low glycemic index diet, respectively. In addition, in a study by Fleischman et al., the majority of patients (14/21, 67%) reported a preference for visits conducted through telemedicine rather than in-person visits [51]. Moreover, another study encouraging the remote management of obese pediatric patients was conducted by Davis et al. [53], which highlights great satisfaction with the intervention, both via telemedicine and via telephone, and both methodologies were highly feasible.

It is worth noting that families who have utilized telemedicine resources have provided positive feedback [40,54].

The use of telemedicine has provided patients with innovative access to health care and physicians with rapid patient assessment, monitoring, and treatment. Several studies have highlighted how these benefits, along with the cost-effectiveness of remote videoconferencing visits compared with in-person visits, have improved the quality of life for patients and their caregivers [40,54].

Telehealth has great potential to ensure that moderately ill patients get the supportive care they need while decreasing their exposure to other acutely ill patients, especially if they are SARS-CoV-2 positive [24,54,55].

A recent study has documented that, while patients are comfortable embracing telehealth, several limitations still exist. Even with new telehealth technology, various barriers to accessing telemedicine include the necessity of having powerful internet connections, software, and equipment. [24,56]. Moreover, research has shown that it is expensive to maintain telemedicine software, especially in rural areas where such equipment can be most beneficial [57].

The professional and ethical challenges that come with web-based health care concern patients and physicians. To start with, patients and their caregivers may be unaware of the telehealth option and may not know how to properly access it; secondly, in their time of need, many people switch back to what they are used to doing and the way they previously interacted with the health care system [24,56]. 

Ultimately, patients and their caregiver may be hesitant to engage in telehealth meetings because of their preference to see a physician in person or their attitude toward technology [24,56].

To summarize, both health care providers, patients, and their caregivers may actually benefit from the adoption of telehealth services, which reduce unplanned hospitalizations, health care service costs, and financial burden for families, as well as guarantee an improvement in caregivers’ satisfaction [54]. However, as previously stated, there remains several limitations of telemedicine offerings and further investigation is needed to confirm the effectiveness of this service [54,58].

## 4. Pediatric Obesity during the COVID-19 Era

Obesity and COVID-19 are pandemics that negatively affect the general health and well-being of children [59]. Firstly, childhood obesity is globally a major long-term public health [60] concern, which has dramatically increased over the past four decades, rising to pandemic levels in United States (U.S.) young people [59,61]. In 2020, the WHO has estimated the number of children under five years of age with overweight or obesity at 39 million [60]. In Italy, according to the latest “OKkio alla salute” report (2019), 20.4% of children are overweight and 9.4% are obese, and the prevalence of obesity is higher in southern regions than in the North [62]. Our country has the highest prevalence of obesity in all of Europe [62]. 

Obesity is a chronic disease influenced by genetic, environmental, and psychosocial factors [59]. Underlying the development of obesity is the breakdown of the body’s energy regulatory system (ERS). The most common cause of obesity in children is an excess of caloric intake in comparison with caloric expenditure, combined with a genetic predisposition. Childhood obesity is associated with various comorbidities involving multiple organs and systems, including type 2 diabetes mellitus, hypertension, nonalcoholic fatty liver disease, obstructive sleep apnea, and dyslipidemia [63].

Along with the obesity pandemic, which has been ongoing for years, another pandemic has been developing since the end of 2019. In fact, in March 2020, the World Health Organization declared COVID-2019 a global pandemic [64]. COVID-19 is a highly infectious disease responsible for significant rates of illness, hospitalization, and death among humans [10]. The virus frequently causes respiratory manifestations such as fever, cough, and dyspnea. Gastrointestinal symptoms are also reported [65,66]. In some cases, a severe infection can develop, which can be fatal, manifesting as acute respiratory distress syndrome (ARDS) and multi-organ failure [65,67]. 

The main risk factors that create a link between obesity and COVID-19 are chronic subclinical inflammation, impaired immune response, and underlying cardiorespiratory disease. In fact, these are proven risk factors for adults, but are also present in obese children and adolescents [68,69]. Furthermore, all the comorbidities found in obese adults can also be observed during childhood and adolescence [68,70]. Zhang et al. [71] have shown how obesity predisposes to high mortality from COVID-19, even in young patients, aged 14 years and older, and, perhaps, it is precisely the high prevalence of obesity among young people that seems likely to shift the mortality age-specific curve in countries where the prevalence of overweight is higher in this group [68,72]. 

Although less frequently compared with the adult population, COVID-19 affects the pediatric age group. According to some studies, the incidence of the disease in the child and adolescent group reaches 5% of confirmed cases, with a slight prevalence in the male sex [73,74]. The pediatric literature has shown that the prevalence, symptoms, and mortality in children with COVID-19 are lower compared with adults. Most cases have mild to moderate infection, 13% are asymptomatic, and 3% have severe disease [75].

The commonly reported COVID-19 symptoms among children and adolescents are slight upper airway symptoms (cough, sore throat, sneezing, wheezing, rhinorrhea, and nasal obstruction), gastrointestinal symptoms (such as diarrhea and vomiting), fever, myalgia, and fatigue [76,77,78,79,80]. Unremarkable alterations, including leukocytosis, leukopenia, lymphopenia, and slight elevations in acute phase proteins, have been the most common laboratory findings [78,80,81,82]. Generally, the radiological abnormalities were less marked than in adults, with most commonly unilateral “ground glass” opacification in the lower lobes, also regarded as mild [73,78,81,82]. The explanations for the reduced severity of COVID-19 in children and adolescents remain partly unanswered. A number of hypotheses have been proposed: lower exposure to SARS-CoV-2 as a result of social isolation and school closures; lower prevalence of comorbidities and smoking exposure compared with adults; a better capacity for lung regeneration [83,84]; absence of the immunosenescence typically found in older individuals, which is a condition characterized by a chronic inflammatory state [83,84,85]; and the fact that children also have a stronger innate immunity response, which is intermediate-lasting, because of increased exposure to viruses and vaccines, which likely leads to early control of infection [68,83,84,86,87,88,89], which is why mortality rates from COVID-19 are lower in countries that administer universal BCG vaccination, compared with those that do not undertake this strategy [68,83,84,90]. Furthermore, infants have a reduced expression of angiotensin-converting enzyme-2 (ACE-2) compared with adults, making the process of virus internalization less efficient [68,84,91,92]. It is interesting to consider that obesity and overweight also appear to be correlated with ACE-2 expression in the lungs; studies in animal models show that rats fed a high-fat diet have increased lung expression of ACE-2, an element that could help explain the greater severity of disease among obese individuals [68,93].

In addition, an inflammatory syndrome called COVID-19 associated multisystem inflammatory syndrome (MIS-C) or “pediatric multisystem inflammatory syndrome (PIMS) may develop in children after infection [94,95]. MIS-C shares some characteristics with Kawasaki disease (KD) and, as in the development of KD, infection is believed to trigger a dysregulated immune response in genetically predisposed individuals. However, although KD and MIS-C share many similarities, they differ in a number of ways. In particular, the complete clinical picture is distinct (coronary artery dilatation is typical in KD, which is not in MIS-C) and the profile of cytokines leading to the inflammatory process appears to be distinct (while MIS-C is driven by IL-6 and IL-8, in KD, IL-1 is predominant) [94].

Major risk factors that increase mortality in adults include age over 65 years, diabetes, hypertension, chronic respiratory disease, and obesity [96,97,98]. To date, the role of comorbidities, such as obesity, in pediatric patients with COVID-19 infection has been poorly investigated. However, because obesity is characterized by chronic systemic inflammation, it is known to be a condition associated with increased production of pro-inflammatory cytokines, such as CRP (C-reactive protein) and interleukin-6 (IL-6) [99]. Elevated levels of these cytokines have also been observed in patients with severe COVID-19 infection [100]. Nevertheless, obesity is also being identified as a dependent risk factor for COVID-19 disease burden [101]. Obese children are likely to experience a more severe COVID-19 disease, including the requirement for respiratory support [102].

The COVID-19 pandemic forced governments to enforce containment measures to reduce the circulation of the virus, thereby evoking dramatic lifestyle changes, with economic, social, and health consequences, and this has especially negatively affected children. As schools closed, children eventually lost the daily access to nutritious food, a safe place to stay, and mandatory physical activity, as well as their social networks and family routines. Pietrobelli et al. [103] showed that, during the lockdown, a group of obese Italian children modified their diet, activity, and sleep habits, exposing them to an increased risk of weight gain. In this research, 41 obese children and adolescents were followed during a three-week shutdown in Verona, with no change in vegetable consumption, and an increase in the consumption of fruit, chips, red meat, and sugar-sweetened beverages; time dedicated to sports activities was reduced by 2.5 h/week and, conversely, time spent sleeping increased by 0.65 h/day; however, the most significant data concerns time dedicated to screen time, which increased by 4.85 h/day [103].

Some studies have observed changes in anthropometric and metabolic parameters that occurred in obese children compared with pre-COVID-19 pandemic [104,105,106,107]. A study conducted on a group of Korean children showed that, within 6 months of social withdrawal and school closure, harmful effects on children’s health were already occurring, in particular weight gain, even in normal-weight children, and vitamin D decrease. Further, the duration of school closure has a significant positive linear relationship with BMI increase and being normal weight does not exclude the risks of gaining weight. Metabolically, a reduction in children’s vitamin D levels was observed following social isolation and school closure during the COVID-19 pandemic. Vitamin D plays a critical role in bone maturation and mineralization and can cause metabolic syndrome when it is deficient [104].

An intervention with physical and nutrition education is needed to promote a healthy lifestyle among children with obesity during the COVID-19 pandemic, in order to mitigate its negative impact on unhealthy weight gain and childhood obesity [107].

The correlation between obesity and infectious diseases, particularly the viral ones, has been a topic of research for years. During the H1N1 influenza epidemic, it was observed that obese patients had a higher risk of developing the disease, a longer intensive care unit (ICU) stay, and higher mortality [108]. This finding is not only particular to the adult population, but is also being demonstrated at the pediatric age, recognizing an altered immune response, especially cellular, to the influenza virus, and an inadequate vaccine response in obese patients [68,109]. Recently, during the COVID-19 pandemic in Canada, obesity was the third most prevalent demographic factor among children admitted to the intensive care unit, behind only those with severe associated disease, immunosuppression, and cancer [68,110].

In one study conducted on 770 patients in New York City, obese patients were found to be more likely to develop fever, cough, and dyspnea, as well as significantly higher rates of ICU admission or death [111]. Data from three different populations confirm in a systematic review that obesity is an independent risk factor for increased severity of COVID-19, including ICU admission [68]. Finally, Yates et al. [112] showed that the risk of acquiring the disease is higher among obese persons.

Besides the well-known clinical burden, the COVID-19 pandemic has also resulted in socioeconomic changes that may have major repercussions for childhood obesity, especially among the poorest [68,113,114]. On that basis, a major study projected the impact of the COVID-19 pandemic on the prevalence of childhood obesity in the United States by proposing several scenarios that include school closures for several months and reductions in physical activity practiced by children and adolescents; both of these items, as has been observed, negatively affect the prevalence of obesity, mutually influencing each other so that the prevalence of obesity increases exponentially [107]. During COVID-19, job losses and layoffs increased, limiting financial resources [59]. At the same time, many communities saw significant increases in food costs [115]. According to the data collected, many households have predominantly purchased long-life, ultra-processed, high-calorie foods that are low-priced, highly palatable, and usable as a comfort item [106,116]. Higher-calorie foods can negatively impact all children, putting them at risk for weight gain [59].

Living with the COVID-19 pandemic has been characterized by stress and sedentariness. The COVID-19 pandemic also affects psychological health through the fear of catching the virus, family concern, social isolation, financial pressure, and information overload [117]. All of these factors can lead to increased levels of stress and anxiety, which in turn will lead to physical health problems, including obesity [118]. A 2015 study by Isasi et al. [119] reported an association between chronic stress and obesity, characterized by a higher caloric intake and lower diet quality. Stress can influence body weight through biological, behavioral, and psychological mechanisms [118,119]. Biological mechanisms include activation of the hypothalamic–adrenal–pituitary axis, leading to the release of cortisol, which can influence body weight by reducing the brain’s sensitivity to leptin and enhancing the reward pathway [120]. Activation of reward centers in the brain, such as the *nucleus accumbens* and dorsal *striatum*, increases the tendency to consume unhealthy as well as particularly palatable foods high in sugar, fat, and sodium [119]. In terms of behavioral mechanisms, stress, in addition to leading individuals to eat greater amounts of junk food, has the potential to lead to reduced physical activity and can also disrupt sleep and lead to a shorter sleep time, accompanied by increased likelihood of obesity [117]. Sleep quality is deteriorating, resulting in unsatisfactory quality and duration, as a response to coping with stress, the suspension of routine activities in the morning, time spent in front of screens, and so on [103]. In this regard, in addition to predisposing to gaining weight and abdominal adiposity, sleep disorders have other health repercussions, such as insulin resistance, worsening diet quality, poor school performance, and sedentariness [121]. Sedentariness, characterized by activities such as watching TV or playing video games, is correlated with changes related to an increased risk of obesity, such as a high consumption of fast food and sugary drinks as well as sleep disorders [122]. A number of articles have spread the assertion that physical activity should be avoided to protect immunity, assuming that exercise reduces the body’s defenses [68]. However, this fact has not been scientifically proven, even among athletes [123]. In contrast, physical activity is important for proper vitamin D formation when performed outdoors [104,124].

Thus, it becomes clear that the harms caused by the COVID-19 pandemic are all closely enmeshed with one another, ultimately leading to significant repercussions on the health of the general population, and particularly the pediatric population.

## 5. Tele-Nutrition in Pediatric Obesity

### 5.1. Before the COVID-19 Pandemic

Health informatics interventions have been shown to improve access to treatment and screening rates of pediatric obesity even before the COVID-19 pandemic [125]. 

Tele-nutrition first emerged in weight management for obese children belonging to rural populations with poor access to tertiary care. Rural residents tended to report a lower likelihood of exercise and higher rates of obesity, heart diseases, and diabetes than their urban counterparts. An initial retrospective review of medical records demonstrated that weight management with tele-nutrition results in improvements in nutrition, physical activity level, and weight in this pediatric population [126]. Shaikh et al. [127] subsequently showed how tele-nutrition in rural environments improves care through breaking down several commonly encountered barriers, such as the lack of local weight-management programs, patient motivation, and family involvement in treatment. Moreover, Irby et al. [128] further observed BMI percentile reductions in 64% of children looked after by telemedicine and 69% of children seen in person, with no significant difference between groups. Treatment drop-out was similar between the two groups, with 30% for telemedicine and 32% for children seen in person. 

The use of telemedicine even in urban areas has shown beneficial effects on obesity with evidence of stabilization or a decrease in BMI z-score. Families showed satisfaction and found their tele-visits to be more convenient than going to the main urban hospital. Although there was no control group for comparison, this study demonstrated the success of implementing telemedicine even in an urban environment [129].

The first clinical trials have thus been designed to evaluate the possible beneficial effects of tele-nutrition. In 2010, a randomized controlled trial (RCT) was conducted, where some families were randomly assigned to perform in-person medical visits or a telemedicine visit. Feasibility and satisfaction levels related to the telemedicine intervention were favorable, especially because this method minimized health care-related travel and increased participation in counseling. However, no significant difference was found between the two groups, in BMI percentile, nutrition, or physical activity behavior in children [130]. In 2012, a randomized pilot study was performed, comparing one group receiving integrated care of in-person visits and telemedicine and the other group receiving only in-person visits. The integrated care model using telehealth showed greater promise for treating children with obesity in terms of long-term BMI reduction [52]. 

Tele-nutrition has evolved over time and additional approaches have complemented tele-visits in pediatric obesity management. The use of text messaging, mobile applications, and wearable devices in supporting healthy eating habits and physical activity has been tested. Although the studies have been small and heterogeneous in nature [131], preliminary results have shown that telephone intervention can be considered an effective aftercare to stabilize outcomes in obese children [132].

Finally, a recent systematic review examines the literature regarding the effectiveness of clinic-based telehealth versus face-to-face modalities to reduce obesity among school-aged children. Ten studies showed a reduction in BMI (primary outcome) and improvement in eating habits, physical activity, and patient satisfaction (secondary outcome). Only five studies showed better outcomes when telemedicine and face-to-face interventions were proposed as combined therapies. All the authors concluded that the use of telehealth in conjunction with face-to-face visits for obesity treatment among children and adolescents is effective [133]. 

The available evidence highlights the efficacy of these tools in improving weight loss, behavioral change, and drop-out rate [52,127,134,135,136]. On the other hand, the results from Davis et al. [130] should be interpreted carefully given the small sample size and lower frequency of counseling sessions compared with the clinical guideline recommendations. Patients and parents showed satisfaction with lifestyle changes, great enthusiasm about nutrition programs, and increased motivation [51,137]. This was reflected in increased adherence to family obesity treatment, particularly among the rural population, and in family involvement in nutrition programs [42,125].

### 5.2. During the COVID-19 Pandemic

During the current COVID-19 pandemic, both health care professionals and patients have been much more accepting of telehealth services, owing to the high risk of the spread of infection among patients and health care professionals at care sites, limited availability of adequate personal protective equipment (PPE), travel restrictions, limited access to hospitals, and interruption of most clinical activities.

A 20-question survey emailed by the Academy of Nutrition and Dietetics (ADA) between 24 August and 11 September 2020 collected responses from 22 registered dietician nutritionists (RDNs) [138]. The responses showed that, prior to the pandemic, 15 hospitals had not provided nutritional care via telemedicine. Instead, during the pandemic, 20 hospitals began providing telehealth nutrition service. The areas in which telehealth was used included nutritional screenings, recommendations for nutrition supplements, nutritional assessment, and nutrition education. 

The questionnaire revealed that mainly telephone calls were used during COVID-19, along with videoconferencing and remote patient monitoring. The RDNs reported that it was difficult to conduct nutritional focused physical exams (NFPEs); on the other hand, they could manage to have a longer assessment time and to look at the patient’s home environment and nutrition habits [138].

In pediatric obesity, nutrition education for the whole family is the first treatment. Telemedicine visits are a great opportunity to get the whole family involved. Using a computer, tablet, or text messages, educational material can be shared and explained during the visit to encourage the patient and all stakeholders to be more active. Another potential benefit of telehealth nutrition care is that it increases access to medical nutrition therapy (MNT) by removing barriers related to limited finances, travel, time, and physical function, especially during COVID-19 [139]. 

To overcome these challenges, telehealth and mobile health technologies have been used alongside standard therapy for pediatric weight management, even before COVID-19, as previously reported.

During the COVID-19 pandemic, two hospitals from New York City exclusively performed telemedicine appointments [114]. The health care providers involved offered nutritional, physical activity, and psychological support through a virtual interdisciplinary tool with simultaneous access to the patient’s medical records. Interestingly, the access rate to pediatric telemedicine examinations before COVID-19 was 55–65%, but during the pandemic it has risen considerably to 76–85%. Clearly, the higher technological availability and the limitations to reach hospitals impacted the access rate [114]. 

O’ Hara et al. reported their experience during the SARS-CoV-2 pandemic, highlighting that families who had experienced telemedicine provided positive feedback. For some patients, telemedicine at home had improved access by reducing travel barriers. They were also enthusiastic to share nutritional information with health care providers. Moreover, patients and parents could include more family members during the visit. These aspects make telehealth a tool for sharing information and providing nutrition education with a greater impact on family habits. The authors concluded that, pre-COVID-19, the financial impact of telemedicine was 10%. On the other hand, having offered 100% telemedicine visits during the pandemic, a different rate table must be established between in-person and telemedicine visits, striking a balance between the two services offered [7]. 

## 6. Tele-Exercise in Pediatric Obesity

### 6.1. Before the COVID-19 Pandemic

During the last two decades, thanks to the improvement of electronic devices and the spread of online technologies, training and exercise practice was implemented through applications, web channels, and online platforms and specific active videogames (called exergames) were also developed to bring exercise training closer to online technologies [140,141]. Before the COVID-19 pandemic, the use of tele-exercise and exergames as home training was high, especially among children with diseases, such as cerebral palsy, bone cancer, and cystic fibrosis, and aimed to limit public exposure, risk of infections, and transport barriers [142,143,144]. In a study conducted among children with cerebral palsy, during an online individualized training program, specialists deduced that virtual reality was effective in cognitive function improvement through levels of attention and increased concentration [142]. In addition, other studies in patients with spastic hemiplegic cerebral palsy showed an improvement in upper limb motor function and an increase in manual strength [145]. Chen et al. [143] proposed an interactive tele-exercise program with wearable devices in children with cystic fibrosis, suggesting that the streaming of sessions is a viable and convenient method to encourage PA practice without cross-infection risks associated with in-person group activity. Finally, Cosano et al. [144] reported benefits on bone health in pediatric cancer survivors through a supervised online training program.

In children with obesity, exergames have been proposed to reduce sedentary behaviors, improve sport participation, and reduce the risk of respiratory and cardiovascular diseases, generally as a tool to increase PA [146].

Exergaming is based on the use of videogames and devices and converts body movements into avatar’s movements on screen [147], offering the possibility of an immersive experience in a realistic three-dimensional setting or a nonrealistic experience with no sensation of being immersed in the virtual world [147]. In this context and directly in a home-based setting, PA is translated into the game activities, favoring the maintenance of physical fitness and of long-term adherence to exercise [148]. Moreover, exergames are perceived to be easy to set up and use, and appear to be safe for every day use [148]. These characteristics are crucial to increase motivation and self-efficacy, as well as the game requests not requiring complex techniques or coordination, and thus allowing sedentary populations to approach this training [148]. 

Systematic reviews and meta-analyses indicate that exergames players can reach light to moderate intensity PA [149,150]. In 2013, a systematic review was performed by Lamboglia et al. to analyze the use of exergaming against childhood obesity [151]. Exergaming was shown to increase PA levels, energy expenditure, maximal oxygen uptake, and heart rate, and to reduce waist circumference and sedentary screen time, supporting individuals to become more active, especially those that experience difficulties or embarrassment during exercise in public, as may be the case with obese youth [152]. 

Other researchers suggested that exergame interventions had significantly positive effects on children’s obesity-related outcomes, body composition, and PA participation. For example, Bethea and colleagues [153] reported an enhancement in children’s cardiovascular fitness after a 30-week of tele-exercise intervention. Calcaterra et al. [154] also reported improvement in body composition, cardiorespiratory fitness, and metabolic profile through a supervised recreational training program of 12 weeks for sedentary obese children, including interactive video game exercises both during the supervised sessions and in a home-based setting. Other researchers found that the exergame treatment effects were related to PA level, BMI, and body fat reduction with specific exergames (e.g., Eyetoy, Kinect Sport) [149,150]. In a study with overweight children, Murphy et al. [155] found that children in the tele-exercise group had a significant increase in total exercise time and VO_2_ peak, and a decrease in body weight compared with control children. However, several studies have reported that exergame interventions had no effect on children’s body composition (body mass index and percent of body fat) and PA levels [156,157] for reasons related to the duration of the program, dropout rates, and the absence of supervision.

### 6.2. During the COVID-19 Pandemic

The World Health Organization (WHO) recommends at least 60 min/day of moderate to vigorous intensity physical activity (PA) engagement for people aged 5–17 to achieve musculoskeletal and cardiovascular health benefits and psychological wellbeing [6]. During the COVID-19 pandemic, PA or exercise restriction at school or in outdoor settings leads to a vicious cycle of sedentary behavior and decreased daily energy expenditure, contributing to weight gain. Moreover, the introduction of containment measures limits PA practice in children and adolescents and could curb the correct age-development and acquisition of motor skills such as coordination, agility, and team-cooperation [121]. In fact, for young people, PA and exercise through movement, music, and energy expenditure have numerous benefits in the psychosocial, physiological, and developmental realms [158,159]. In light of this, the implementation of recreation and games as well as programmed PA at home becomes of primary importance. In fact, the WHO Regional Office for Europe has provided basic information on the relevance of PA during self-isolation and recommended ways to be active, one of which has been following “online exercise classes”. The Centers for Disease Control and Prevention has also endorsed “workout videos”. The National Health Service has recommended online exercise classes in order to improve and maintain mental health. Technology-supported intervention approaches use different support mechanisms, such as tailored PA advice, goal setting, feedback, PA tracking, tele-counselling, online resources, and online social support [160,161].

For these reasons, the possibility to maintain an active lifestyle and to practice sport diminished and most of the options for group/peer supportive exercise were with online classes, web channels, mobile applications, and exergames [161,162]. In fact, prior to the COVID-19 pandemic, technology-supported approaches to promote PA were shown to be effective in increasing PA levels, walking, and energy expenditure [163,164,165]. These interventions use different support mechanisms, such as advice, goal setting, feedback, PA tracking, tele-counselling, online resources and online social support [160], and online exercise classes. In pandemic times, these exercise modalities were particularly exploited as they are cost- and time-efficient and can be flexibly used in an environment with no infection risk. A recent multinational survey with 10,433 participants examined the attendance in digital home training programs and found that 68% of participants reported such interest.

In children with type 1 diabetes, during the COVID-19 lockdown, Calcaterra et al. [166] proposed “CoVidentary”, an online exercise training program to reduce sedentary behaviors. The outcomes obtained by the exercise sessions showed that it is possible to reduce a sedentary lifestyle, but not to achieve the optimal level of recommended PA for children. In particular, this study concluded that a short duration training session developed through an online platform is more effective to encourage participation in a self- administrated exercise. Furthermore, a reduction in PA and exercise practice was shown in obese children, as well as augmented time spent on television and mobile phones. For these reasons, the tele-exercise and exergaming interventions could be important tools to reduce sedentary behavior and maintain an active lifestyle. Calcaterra et al. [162] proposed a different modality of exercise such as active breaks, playing with pets, balloon games, cleaning races, and music parties that could be performed at home and without specific equipment in safe conditions and without the risk of infections. Moreover, Staiano et al. [8] proposed the GameSquad intervention, a 24-week home-based exergaming intervention tailored for children and adolescents with obesity to investigate adherence, promote self-efficacy, and reduce perceived barriers. This study reported a reduction in BMI z-score and an improvement in cardiometabolic health (systolic and diastolic BP, total cholesterol, and LDL-cholesterol) among children with overweight and obesity. These results expand upon a home-based exergaming trial that effectively reduced BMI z-score and body fat over a 6-month period [167]. Furthermore, the GameSquad intervention obtained excellent adherence for children’s exergaming (94% over 24 weeks) by employing social support, including regular video chats with fitness coaches and a gaming curriculum and step tracker to motivate children’s PA. Telehealth directly involves the child in communicating, monitoring, and counselling on exercise.

To limit the spread of pediatric obesity, curricular sport and physical education in schools typically had a fundamental role and represents the largest and effective youth PA intervention worldwide [168]. Although regular in-person physical education programming is not without challenges [169], school closures due to COVID-19 created a new host of obstacles, especially in the obese population. In many countries, physical education shifted to virtual learning platforms [170], and physical education teachers and administrators were swiftly required to deliver robust virtual programs without adequate training and provision of appropriate teaching and learning resources. Online learning could produce bias, especially in youths owing to the unequal access to technology, adult supervision, and support, as well as lack of sports equipment and physical space to participate in online physical education [171]. Additional inequities are presented for youth with obesity who are particularly dependent on school physical education for PA engagement, and face barriers to being physically active in home environments [172]. Despite all these factors, if designed appropriately, online physical education may have the potential to reduce health disparities related to inequitable opportunities for PA engagement [173], reducing the distance between children with obesity and sport facilities, transport barriers, as well as the cost and the risk of contagiousness. For these reasons, tele-exercise surely needs further improvements, but becomes a valid tool in this unique period to help children with obesity to maintain an active lifestyle, participate in adapted activities, and share time and experience with their peers in a protective environment.

## 7. Future Prospective after the COVID-19 Pandemic

Tele-nutrition has been demonstrated over time to have good benefits in the management of obesity, assuming the child is fit and the service provided is of comparable quality to in-person care provided [42]. The effectiveness of tele-nutrition at reducing BMI and improving adherence to treatment assumes the incorporation of telemedicine into the care of pediatric patients with obesity. It has also been shown to be cost-effective by minimizing travel expenses [125]. This is a crucial aspect, which could promote early initiation of effective treatment for low-income families and long-term adherence in the future.

However, some prerequisites are needed to overcome the few inherent limitations of this method. Tools for measuring auxological parameters and monitoring obesity-related complications should be accurate and used continuously during all visits. Caregivers should play an “active part” during the visit and be able to make accurate measurements, but they cannot perform the medical evaluation of the patient.

Tele-exercise has been shown to increase PA levels, energy expenditure, maximal oxygen uptake, and heart rate, and to reduce waist circumference and sedentary screen time, supporting individuals to become more active and improve self-efficacy and cognitive function, especially in subjects that experience difficulties or embarrassment during exercise in public. If appropriately designed, tele-exercise could also be useful to improve adherence to long-term exercise. Finally, tele-exercise has been shown to reduce public exposure; risk infection and transport barriers; and directly involve children in the communicating, monitoring, and counselling on exercise.

Using these approaches has proven to be an important way to share information within the team and has allowed multiple obesity specialists to attend visits [51]. 

**Figure 3:**
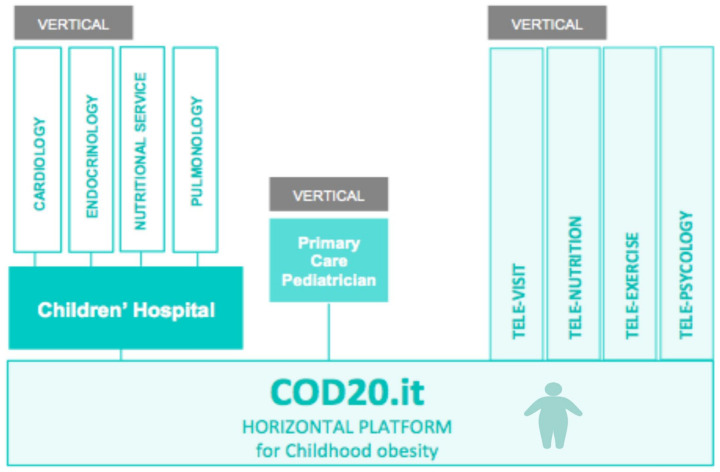
Proposal of virtual platform, using the COD20 model [25], as a novel approach to childhood obesity surveillance (created with BioRender.com, accessed on 16 October 2021).

The COD19 and COD20 model proposed by Zuccotti et al. [25] could provide a template of the virtual hospital for remote clinical intervention that is also appropriate for children with obesity. COD20, a single platform with horizontal technology that shares the functionality with all actors and offers the possibility of verticalization for additional interventions and customizations, may be a useful tool for an integrated approach to obesity care, providing video-consultations and offering the simplification of the hospital visit booking process (Figure 3). 

Maintaining communication between specialists is a prerequisite for encouraging shared decision-making, as well as sharing information with the patient being key to promoting compliance. This could be a prerequisite for closer patient monitoring and increasing patient expectations and trust in obesity specialists.

**Figure 4:**
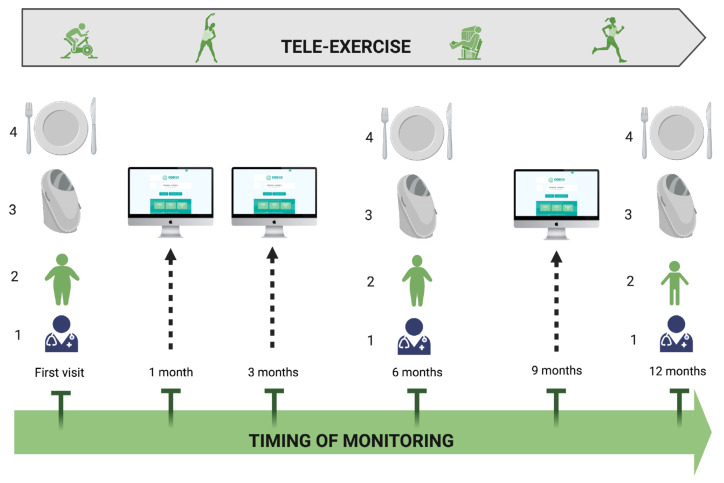
Integrated care model for childhood obesity, combining telehealth and face-to face visits. Face-to face visit is proposed at the first evaluation and at 6- and 12-month follow-up; clinical evaluation (1), body weight control (2), body composition evaluation (3), and nutritional counseling (4) are included. At 1-, 3-, and 9-month follow-up. Tele-consultation for monitoring of weight, dietary intake, and physical activity is provided. During follow-up, a supervised exercise training program is remotely delivered three times per week in a 30–45 min session (created with BioRender.com, accessed on 16 October 2021).

Using a patient–specialist video consultation service, such as COD20 [25], an integrated programme, combining telenutrition, tele-exercise, and face-to face visits, could be proposed for childhood obesity management; Figure 4.

Further studies could investigate the utility of telemedicine combined with in-person visits to address their limitations. If existing care practices are revised, then access to care for patients and the skills of involved professionals should be considered [139]. Long-term studies will document the effectiveness, accessibility, and feasibility of telemedicine in pediatric obesity care as a successful tool in non-pandemic management as well [137]. 

## 8. Conclusions

Childhood obesity and COVID-19 are international pandemics. The coexistence of the two diseases and changes in the bioecological environment have put children and adolescents at increased risk for developing obesity and exacerbating the severity of this disorder. Therefore, during the pandemic period, health care providers, dealing with obese or overweight children, should manage the whole health status monitoring, in particular, the nutrition area.

Tracking any comorbidities associated with obesity; ensuring that their dietary treatment is not interrupted; providing information to the family while respecting the specifics of the condition; and determining, when necessary, referral to hospital units appropriate for the care of obese children and adolescents are specific actions for the use of telemedicine in childhood obesity management [68].

Tele-exercise has the promise to provide a supervised group exercise experience while mitigating the potential problem of cross-infectivity. The tele-exercise platform allows for flexible scheduling and is accessible from home, bypassing transportation barriers. Incorporating remote monitoring was essential for instructors to adjust the exercise prescription based on the participant’s performance. In the frame of a home-based approach during a period of global emergency, online platform and exergames may be particularly suited to implement rehabilitative interventions, ensuring an adequate level of PA at home with long-term adherence to exercise for both recreative and fitness purposes.

The use of telemedicine offers innovative access to health care, ensuring the supportive and comprehensive care of children and their families, guaranteeing a systematic assessment of their health and biopsychosocial needs, which is critical for reducing the negative impact of obesity and COVID-19 [68]. Even in the post-COVID-19 pandemic, the use of telemedicine could be a prerequisite for closer patient monitoring and increasing patient satisfaction.

## Data Availability

Not applicable.
